# Screening for Normal Pressure Hydrocephalus on Head CT Using Automated Callosal Angle Assessment

**DOI:** 10.3390/tomography12060083

**Published:** 2026-06-03

**Authors:** Sennett Yang, Jazza Jamil, Diep Nguyen, Hannah Murphy, Emily Foldes, Jacob J. Knittel, Maddie Muenzer, Clay M. Oliver, Raza Mushtaq, Justin L. Hoskin, Matthew T. Borzage, Kevin S. King

**Affiliations:** 1 School of Medicine, Creighton University, Phoenix Campus, Phoenix, AZ 85004, USA; sennettyang@creighton.edu (S.Y.); clayoliver@creighton.edu (C.M.O.); 2 Department of Neuroradiology, Barrow Neurological Institute, St. Joseph’s Hospital and Medical Center, Phoenix, AZ 85013, USA; jazzajamil@yahoo.com (J.J.); emily.foldes@barrowneuro.org (E.F.); rmushtaq@sniweb.net (R.M.); 3 Department of Radiology, St. Joseph’s Hospital and Medical Center, Phoenix, AZ 85013, USA; diepnguyen@creighton.edu (D.N.); hmurphy10@gmail.com (H.M.); jacobknittel@creighton.edu (J.J.K.); 4 Arizona State University, Phoenix, AZ 85004, USA; mmuenzer61@gmail.com; 5 Department of Neurology, Barrow Neurological Institute, St. Joseph’s Hospital and Medical Center, Phoenix, AZ 85013, USA; justin.hoskin@commonspirit.org; 6 Fetal and Neonatal Institute, Division of Neonatology, Children’s Hospital Los Angeles, Keck School of Medicine, University of Southern California, Los Angeles, CA 90027, USA; borzage@usc.edu; 7 Alfred E. Mann Department of Biomedical Engineering, University of Southern California Viterbi School of Engineering, Los Angeles, CA 90089-1111, USA; 8 Department of Regulatory and Quality Sciences, University of Southern California Alfred E. Mann School of Pharmacy and Pharmaceutical Sciences, Los Angeles, CA 90033, USA

**Keywords:** normal pressure hydrocephalus, callosal angle, computed tomography, automated image analysis, dementia screening

## Abstract

**Background/Objectives:** Normal pressure hydrocephalus (NPH) is a treatable cause of gait impairment and fall risk in older adults, yet it remains frequently underdiagnosed. This study aimed to validate an automated measurement of the callosal angle, a recognized imaging marker of NPH, adapted for use on routine head computed tomography (CT). **Methods:** We performed a retrospective analysis of 198 patients with probable NPH, confirmed by gait improvement following lumbar tap test, and 198 age- and sex-matched controls presenting with headache and negative head CT findings (mean age 74 ± 7 years; 60% male in both groups). Manual callosal angle measurements were independently obtained by trained residents and reviewed by neuroradiologists. Automated and manual measurements were compared using intraclass correlation, and diagnostic performance was assessed across threshold values. **Results:** Automated callosal angle measurements demonstrated strong agreement with manual measurements (ICC = 0.90). Using an automated callosal angle threshold of <90°, diagnostic accuracy was 84.1%, with sensitivity of 90.4% and specificity of 77.8%. Optimization to a 95° threshold yielded an accuracy of 85.9%, with both sensitivity and specificity of 85.9%. The area under the receiver operating characteristic curve was 0.915 (95% CI, 0.897–0.933). **Conclusions:** Automated callosal angle assessment on routine head CT provides reliable and scalable detection of NPH, supporting its use as a screening tool to facilitate earlier diagnosis and treatment of a potentially reversible cause of dementia.

## Simple Summary

Normal pressure hydrocephalus is a treatable cause of gait impairment and cognitive decline in older adults, yet it is frequently underdiagnosed. A key imaging marker is the callosal angle, traditionally measured manually on MRI. In this study, we validate an automated callosal angle measurement applied to routine head CT scans. Automated measurements closely matched expert manual assessments and accurately identified patients with normal pressure hydrocephalus. This approach enables scalable screening using widely available CT imaging and may support earlier diagnosis and treatment of a potentially reversible cause of dementia.

## 1. Introduction

Normal pressure hydrocephalus (NPH) is a subtype of hydrocephalus categorized by ventriculomegaly without an increase in mean intracranial pressure. NPH predominantly leads to gait and balance disorders but in many can lead to a triad with urinary incontinence and cognitive decline, which is known as Hakim’s triad [1,2]. NPH can be treated with the surgical placement of a ventricular shunt, with efficacy recently affirmed in a randomized trial [3].

The severity and difficulty of diagnosing NPH lead to a need for an effective early screening tool. NPH is difficult to diagnose, as ventriculomegaly and associated clinical symptoms are nonspecific [4]. Ideally, NPH should be diagnosed early as this has been shown to improve shunting response and long-term recovery [5,6,7].

Imaging can play an important role in providing an early indication of potential NPH that may not have been considered. Diagnostic imaging biomarkers such as Evans’ index and the callosal angle (CA) have been shown to accurately identify NPH [8]. Systemic diagnostic panels that utilize a number of biomarkers have shown an ability to identify NPH with 100% sensitivity and 96% specificity [9]. Unfortunately, applying these measures takes time and cannot be conducted on every patient. Automated assessments performed on MRI have been shown to help identify NPH [10], but many patients presenting with acute symptoms of altered mental status or falls obtain head CT. As a result, we have developed a method that utilizes clinical computer tomography (CT) scans to facilitate an NPH diagnosis by automating CA measurements, which has been shown to have a greater specificity for NPH than other suitable biomarkers such as Evans’ index [11].

This retrospective study aims to validate the efficacy of a published automated MRI CA measurement algorithm redesigned using CT images. Using published CA cutoffs, we aim to test the ability of our program to differentiate NPH ventriculomegaly from control patients. We developed this algorithm using 915 head CT images, of which 313 were tap-test-responsive patients with NPH treated at the multidisciplinary Barrow Neurological Institute Normal Pressure Hydrocephalus Clinic.

## 2. Materials and Methods

### 2.1. Patient Population

**Figure 1:**
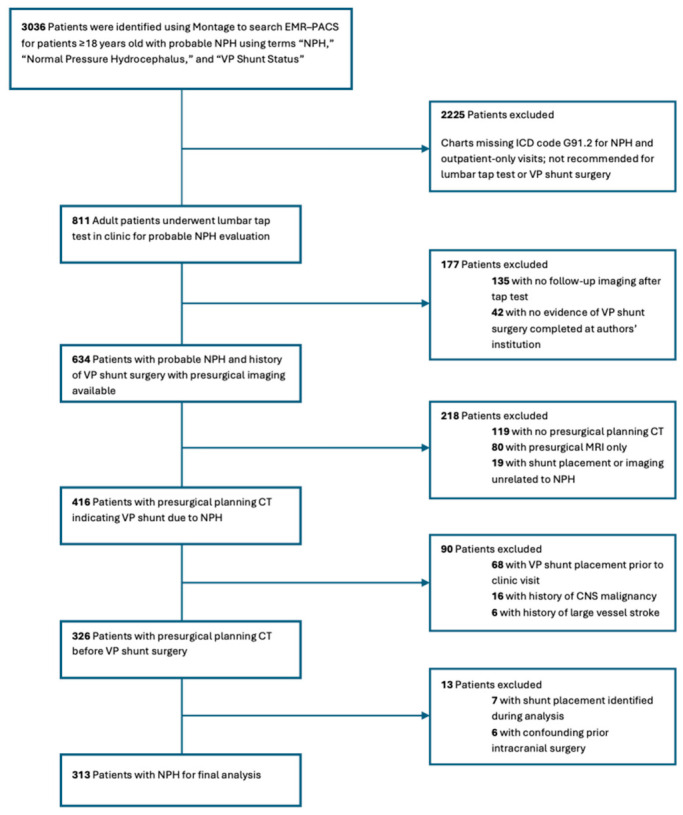
Patient inclusion and exclusion criteria. EMR-PACS = electronic medical record-PACS; ICD = International Classification of Disease; CNS = central nervous system.

Following approval from the Institutional Review Board at St. Joseph’s Hospital and Medical Center, we identified 313 consecutive patients with probable normal pressure hydrocephalus (NPH) from the Barrow Neurological Institute NPH Clinic who showed gait improvement post-cerebrospinal fluid (CSF) tap tests. Patients were included if they were at least 18 years old, had probable NPH, had subjective and objective improvement following the tap test, and were identified using search terms such as “NPH,” “normal pressure hydrocephalus,” or “VP shunt status”. Pre-surgical planning CT scans were obtained and archived following a retrospective review of electronic medical records conducted using Montage (Montage Healthcare Solutions Inc., Philadelphia, PA, USA), and a de-identified dataset was created with patient informed consent, which yielded 3036 patients. Subjects were excluded if they were missing ICD code G91.2 for NPH, if lumbar tap tests or ventriculoperitoneal (VP) shunt surgery were not recommended, or if outpatient visits were conducted outside our institution. Of these patients, 811 with probable NPH underwent a tap test. From these, 177 were missing post-tap-test imaging or lacked VP shunt surgery completion at our institution, which would indicate loss to follow-up, care provided outside our institution, or a negative tap test results. Further exclusion criteria included those who were missing pre-surgical head CT and instead had pre-surgical MRI, had shunt placement prior to their clinic visit, had shunt placement unrelated to NPH, had a history of CNS malignancy or large-vessel stroke, or had evidence of prior intracranial surgery (Figure 1).

Diagnosis of NPH was made following clinical guidelines, including the presentation of clinical features of probable NPH and gait disturbance with improvement following the withdrawal of 30–40 mL of CSF. Patients were rigorously evaluated before lumbar puncture for potential comorbidities and confounders, including assessment by a gait and balance disorder neurologist. A lumbar puncture was offered only when the patient’s history and examination were consistent with NPH in addition to imaging findings. After the tap test, physical therapy evaluation results were reviewed at a follow-up appointment, and VP shunt placement was offered only when a clear and consistent benefit to walking was observed. Candidates were referred to a neurosurgeon for independent history, examination, imaging review, and tap test review; surgery was performed only when the same conclusion was reached. Patients were additionally assessed for Alzheimer’s and Parkinson’s disease by gait experts, with referral for formal cognitive evaluation when indicated. Only patients who underwent shunt surgery at our institution were included in this study.

Images were evaluated for ventricle-to-sulcal discordance (i.e., disproportionately enlarged ventricles, tight or effaced sulci at the high convexities, and enlarged Sylvian fissures) to screen for non-NPH ventriculomegaly. Control images were obtained from 602 emergency department patients presenting with headache who underwent CT imaging and were negative for any acute intracranial findings.

### 2.2. Head CT Imaging Technique

CT data were acquired across multiple scanner platforms from different vendors; however, all images used for analysis were reconstructed as 1.2 mm thin-slice datasets using a high-spatial-frequency bone algorithm to maintain consistency for callosal angle measurement.

### 2.3. CSF Tap Test Protocol

From our probable NPH cohort, patients underwent gait testing preceding and following the CSF tap test protocol. The CSF tap test was considered positive if subjective and objective measures of gait and balance improved within 24 h after the tap test.

### 2.4. Manual CA Measurement

**Figure 2:**
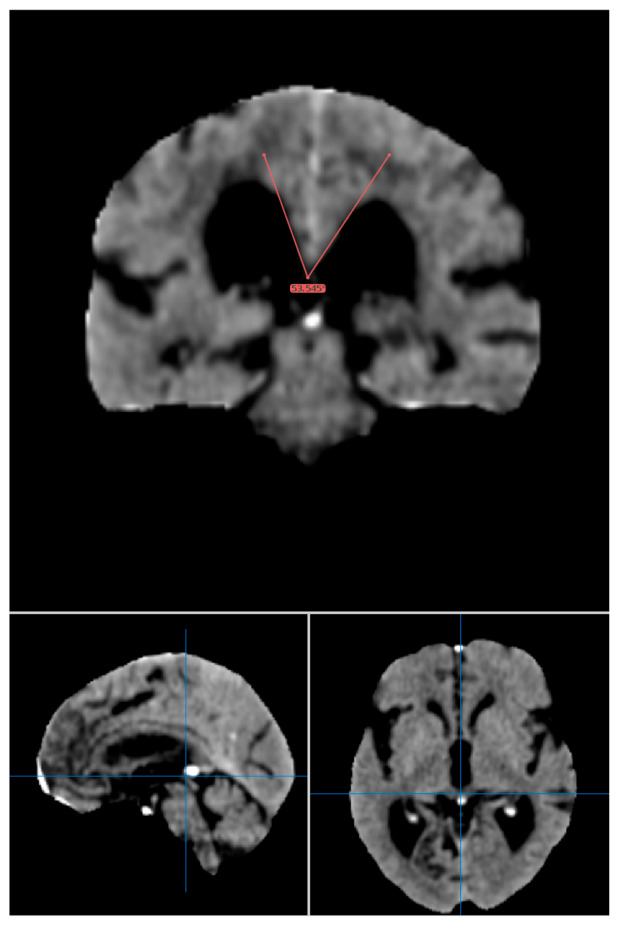
Representative image for manual CA measurement. The upper panel demonstrates the manual measurement of the CA (red lines) on a coronal slice at the level of the posterior commissure. The lower panels display the corresponding sagittal (**left**) and axial (**right**) localizer views, where the blue crosshairs indicate the reference lines used to standardize the coronal orientation.

Manual CA measurements were made for the final 915-patient cohort using established methods [12,13]. After identifying a midsagittal plane, creating a plane through the anterior commissure and posterior commissure, creating a coronal plane perpendicular to the plane at the level of the posterior commissure, and drawing 2 straight lines on the coronal image along the medial walls of the right and left lateral ventricles, the resultant angle between these lines was measured. Manual measurements were made for each image by two second-year radiology residents and one trained second-year medical student with final review by two board-certified neuroradiologists (K.S.K. and R.M) (Figure 2).

### 2.5. Automated CA Measurement

**Figure 3:**
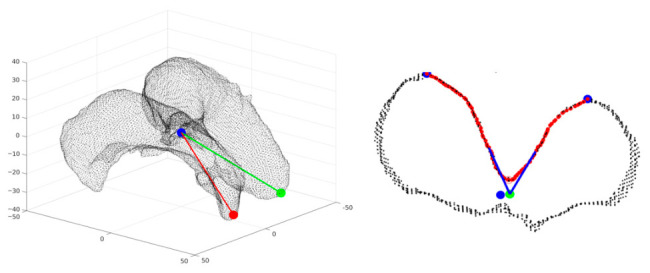
Post-processed segmented lateral ventricle (**Left**) and visual representation of a coronal slice with automated angle measurement using selected points along the lateral ventricles (**Right**). (**Left**) The central blue point defines the centroid, while the red and green points represent the most anterior boundaries from the centroid. (**Right**) The red trajectories illustrate the contours of the medial ventricular walls. The blue lines represent the first-order linear regressions fit from which the final automated CA is calculated.

Automated CA measurements were performed using Matlab (R2020A; MathWorks). The total 915-patient cohort was used to refine and adjust the automated measurement, including pitch angle selection and linear correction. The age- and sex-matched evaluation subset (198 NPH and 198 controls) was drawn from this same cohort. The algorithm used to analyze the ventricles begins by establishing an axial reference plane based on the centroid of the lateral ventricles and the most distal points of the left and right anterior horns. A coronal reference plane is then generated perpendicular to this axial plane at the centroid and subsequently tilted backward to form an oblique orientation. The ventricles are then sectioned along these planes, and the angle between the medial walls of the lateral ventricles is calculated. This coronal plane was shifted anteriorly and posteriorly to determine the optimal location that decreases error compared to manual measurements (Figure 3).

### 2.6. Parameter Optimization

The reference plane’s pitch was systematically varied from −45° to +85° in 5° increments (27 candidate orientations). For each pitch, an automated CA was computed across the development cohort and compared with manual CA measurements; the pitch minimizing the difference was selected. Residual systematic differences between automated and manual values were then corrected by linear regression.

### 2.7. Statistical Analysis

To evaluate the performance of the automated CA measurement algorithm against manual CA measurements, we conducted several statistical analyses using JMP statistical software version 19 (JMP Statistical Discovery, LLC). Utilizing JMP’s Fit Model function, a regression model analysis was performed to assess the correlation between automated and manual CA values. The regression model derived was used to tune and correct the automated measurements, improving the agreement between methods. Receiver operating characteristic (ROC) curves and area under the curve (AUC) values were generated using JMP to evaluate the diagnostic performance of the automated CA measurement. The sensitivity, specificity, and accuracy of the automated CA measurement for NPH detection were calculated using a previously published threshold of <90°. From these results, NPH Radscale scores were measured for false positives and false negatives. This was to verify whether our algorithm had failed or if there were missed NPH cases among controls and clinically diagnosed NPH cases without characteristic imaging. Further assessment of the test performance of the automated CA for identifying probable NPH status was conducted using the Model Classification Explorer to determine the optimal threshold cutoffs that maximize both sensitivity and specificity, while minimizing the rate of misclassifications. Bland–Altman plots were created to visualize the agreement between manual and automated measurements (automated subtracted from manual measurements), as well as between reviewers. The intraclass correlation coefficient (ICC) was calculated to assess agreement between manual and automated measurements, and between reviewers.

## 3. Results

### 3.1. Patient Cohort

In total, 915 head CT images were collected, 602 from controls with normal CT findings and 313 from patients with suspected NPH, diagnosed via showing quantitative gait improvement after lumbar tap tests. After age and sex matching, the sample included 198 NPH and 198 control patients, with an average age of 74 ± 7 years in both groups. The sex distribution was balanced, with 60% men and 40% women in each cohort (Table 1).

### 3.2. Automated Callosal Angle Measurement Accuracy

**Figure 4:**
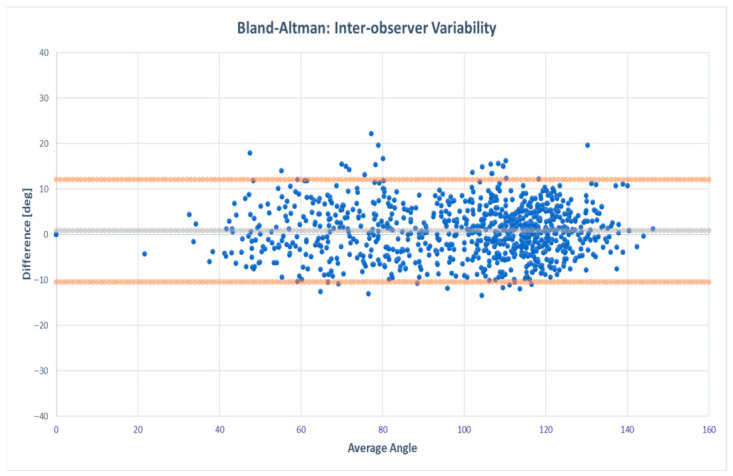
Between manual reviewers, the mean bias was 0.85° (gray line), with 95% limits of agreement from −10.40° to 12.10° (orange lines), and an intraclass correlation coefficient (ICC) of 0.97 for total patient dataset.

**Figure 5:**
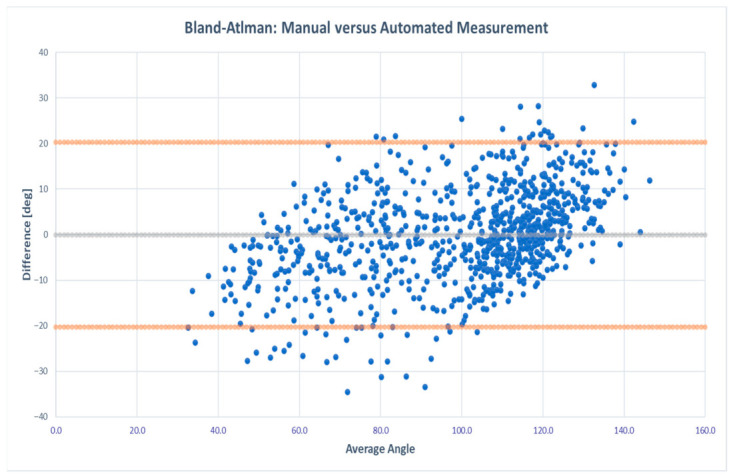
Comparison between the manual and automated measurements (automated measurements were subtracted from manual measurements) revealed a mean bias of 0° (gray line), with 95% limits of agreement of −20.25° to 20.25° (orange lines) with an intraclass coefficient of 0.90 for total patient dataset.

The automated method for measuring the CA on CT scans demonstrated high reliability and diagnostic accuracy for identifying NPH. Comparison between two manual reviewers showed a slight measurement bias of 0.85°, with 95% limits of agreement between −10.40° and 12.10°, and an intraclass correlation coefficient (ICC) of 0.97, indicating excellent interrater reliability. Comparing automated to manual CA measurements revealed 95% limits of agreement from −20.25° to 20.25° and an ICC of 0.90, reflecting strong agreement between methods (Figure 4 and Figure 5).

### 3.3. Diagnostic Performance

**Figure 6:**
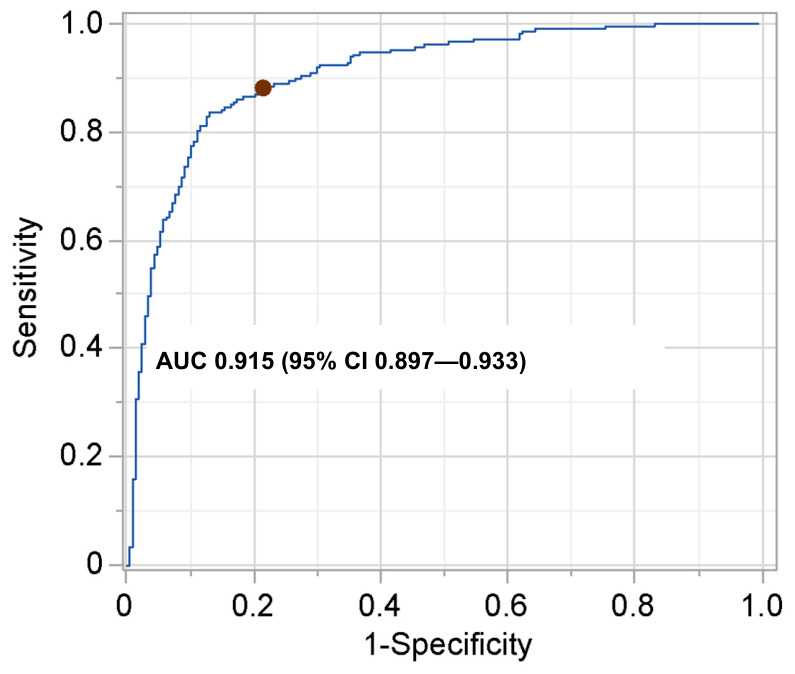
Receiver operating curve and area under the curve (AUC) for automated calculation of the callosal angle.

**Figure 7:**
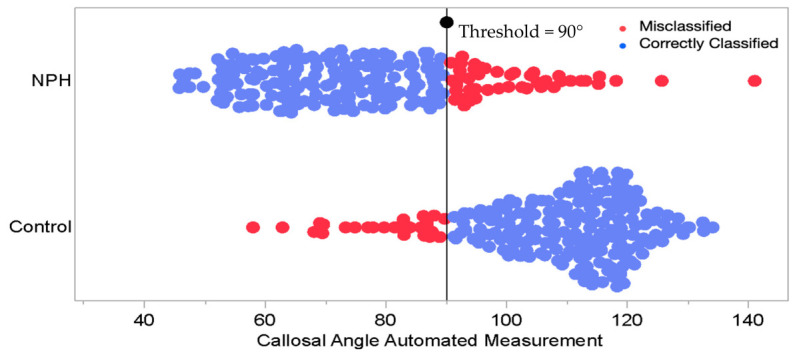
Automated CA measurements for normal pressure hydrocephalus (NPH) vs. control groups. A threshold of 90° was previously established as a reliable cutoff point for NPH screening.

ROC analysis demonstrated an AUC of 0.915 (95% CI: 0.897–0.933), confirming excellent discriminative performance of the automated CA measurement in distinguishing NPH patients from control patients. Using a previously established threshold of 90° for the automated method, the accuracy of our program was 84.1%, with a sensitivity of 90.4% (95% CI: 85.5–93.8%) and a specificity of 77.8% (95% CI: 71.5–83.0%). Optimizing the automated threshold to 95° improved accuracy to 85.9%, with a sensitivity of 85.9% (95% CI: 80.3–90.0%) and a specificity of 85.9% (95% CI: 80.3–90.0%). For this optimized threshold, NPH Radscale scores were measured by a neuroradiologist to evaluate our false negatives and positives (Table 2 and Table 3, Figure 6 and Figure 7).

## 4. Discussion

To our knowledge, this is the first report of an automatic assessment of CA from clinical CT scans compared with manual assessments in a large cohort. We undertook this work as we believe it greatly increases the number of brain scans available for screening. The prevalence of NPH is not well known, but prior work has shown that up to 12.5% of community-based elderly cohorts may have imaging features concerning for NPH [2]. We believe that automated tools like this one can serve a meaningful role in helping to flag these cases for further evaluation so that we can help more people suffering from this condition. Further, our results on this CT screening are in line with values that have been reported for MRI. Borzage et al. found an AUC of 0.87 and Lee et al. applied an AI-based volumetric analysis that included the CA and achieved an AUC of 0.936 on an unseen test set, closely mirroring manual CA-based classification (AUC 0.938) [14]. Our findings, with an AUC of 0.915 and ICC of 0.90 between manual and automated CAs, are comparable and reinforce the feasibility of using automated CA tools in CT-based workflows (Figure 1, Figure 2 and Figure 3).

This study demonstrates that automated measurement of the callosal angle on CT imaging can reliably and accurately distinguish patients with idiopathic NPH from matched controls. In an age- and sex-matched cohort of 198 NPH and 198 control patients, the automated CA analysis achieved excellent discriminative performance, with an area under the ROC of 0.915 (95% CI: 0.897–0.933). In spite of the presence of false positives and negatives at the optimized cutoff of 95°, analysis of corresponding NPH Radscale scores provided additional context for these findings. Most false negatives exhibited intermediate Radscale scores between four and eight, reflecting incomplete or borderline rather than definitive NPH morphology of the disease. Likewise, most false positives demonstrated mildly elevated scores within the same range [15]. Further, 3 of 28 false negatives were found to have Radscale scores < 4, indicating that these patients would have been diagnosed mainly on clinical grounds and positive tap test rather than imaging morphology. These findings indicate that modest reductions in sensitivity and specificity are influenced by inherent ambiguity in borderline imaging cases, where radiographic markers do not clearly distinguish control from NPH states. Therefore, the observed performance at the optimized threshold (sensitivity of 85.9% and specificity of 85.9%) likely reflects the continuum of imaging manifestations rather than systematic over- or under-detection by the automated method. This automated tool would then be useful to help direct which patients would benefit from further verification with manual approaches like the NPH Radscale or further clinical assessment by specialty physicians with experience with NPH. Although tap test responsiveness is not a perfect reference standard for NPH, multidisciplinary clinic review minimized the likelihood of including patients with alternative diagnoses such as Alzheimer’s disease or vascular cognitive impairment. Some residual misclassification cannot be excluded, but any non-NPH patients in the NPH group would likely lower apparent performance—as they would be less likely to show CA narrowing—meaning that our estimates may be conservative rather than inflated.

Manual CA thresholds have traditionally centered near 90°, particularly in MRI-based studies that established CA as a robust discriminator of NPH [16,17,18]. However, some investigators have proposed higher thresholds; for example, Miskin et al. suggested that a cutoff closer to 100° may improve diagnostic performance when the CA is derived from volumetric MRI [10]. These discrepancies highlight that the optimal threshold is not universally agreed upon and likely depends on imaging modality, measurement technique, and patient population. In our study, use of a 90° threshold for automated CA measurements yielded an accuracy of 84.1%. A data-driven threshold of 95° improved diagnostic accuracy, suggesting that threshold optimization may be modality-specific and that higher cutoffs may better accommodate CT-based variability (Table 2). Importantly, our group’s prior work showed that approximately 12.4% of cognitively normal older adults in the ADNI cohort exhibited at least one CA < 90°, underscoring that CA values near traditional cutoffs must be interpreted with caution [2]. Although a threshold of 95° optimized accuracy, since we are proposing this as a screening tool to flag cases for further evaluation with more precise assessments, use of the more sensitive threshold of 90° seems justified to us.

Interrater reliability in our study was excellent (manual ICC = 0.97), and agreement between manual and automated CA measurements (ICC = 0.90) was comparable to previously published inter-method assessments (Figure 1 and Figure 2). To create the inter-method Bland–Altman graph, automated measurements were subtracted from the manual measurements to generate differences. The graph shows that, compared to the manual measurement, the automated measurement tended to underestimate at higher extreme angles and overestimate at lower extreme angles. This degree of approximation at the extremes was permissible as angles further from the threshold were less likely to be misclassified. Borzage et al. reported identical ICCs of 0.97 (manual) and 0.90 (manual vs. automated), while Lee et al. found ICCs of 0.973 in NPH patients and 0.875 in controls. A meta-analysis by Park et al. further confirmed high reproducibility, with a pooled correlation of 0.92 (95% CI, 0.82–0.96) across studies, supporting the clinical reliability of automated CA measurement [2,11,14]. These data suggest that automated CA measurement offers near-expert-level reproducibility, a finding of clinical significance for high-throughput screening or when expert interpretation is unavailable.

We focused on the CA as a single biomarker because it offers higher specificity for NPH than other established metrics and is geometrically well-defined for automated CT measurement, providing a foundation that future multivariate pipelines can build upon.

Nonetheless, diagnostic misclassification persists. At the optimized threshold of 95°, 14.1% of patients with NPH (28/198) were misclassified as controls, while 14.1% of controls (28/198) were incorrectly flagged as NPH patients (Table 2). This underscores a central limitation of the CA as a univariate biomarker. Prior work has shown that combining the CA with other features, such as Evans’ index, disproportionately enlarged subarachnoid space hydrocephalus (DESH) patterns, temporal horn diameter, and high-convexity tightness, improves classification performance significantly [8,19,20]. For instance, Kadaba Sridhar et al. demonstrated that a CT-based morphometric pipeline including CA, Evans’ index, and maximum eccentricity of the lateral ventricles achieved AUCs exceeding 0.98 for distinguishing NPH patients from controls [20]. Gholampour et al. similarly reported that automated feature extraction from brain morphology, including splenial and anterior CAs, enhanced diagnostic fidelity in hydrocephalus [16]. Automated assessment of the ventricle-to-subarachnoid ratio, which is meant to assess ventricle-to-sulcal concordance, has also shown an AUC of 0.99 [21]. This raises interesting questions about whether there are different stages or different types of NPH with different imaging features, not all of which may include a narrowed CA.

From a technical perspective, our study validates the use of automated CA tools on CT, with performance metrics that match those reported in recent AI-based MRI pipelines [14,16,20]. This is particularly relevant for the assessment of patients presenting to urgent and emergent care clinics with altered mental status or falls, where CT is often acquired. Moreover, the consistent sex distribution and precise individual age matching in our cohort enhance the generalizability of our findings, which is a frequent limitation in retrospective NPH datasets.

Finally, the integration of CA measurement into AI-enhanced, multimodal diagnostic systems is endorsed by the 2025 ACR Appropriateness Criteria for dementia workup, which emphasizes CA ≤ 90°, Evans’ index > 0.3, DESH features, and periventricular white matter changes as core imaging markers for NPH [17]. The growing literature supports a shift toward fully automated diagnostic pipelines that combine these metrics using machine learning algorithms such as gradient boosting and XGBoost [14,20,22].

For this tool to translate into clinical impact, it must integrate into the existing radiology workflow without adding interpretive burden. The algorithm could run as a background analysis on routine head CT, delivering results to the radiologist as a structured field. Studies below the screening threshold could be flagged to prompt consideration of NPH and a recommendation for clinical correlation in patients without prior workup. Prospective implementation studies will be needed to refine thresholds and assess downstream clinical impact.

### 4.1. Future Directions

Several avenues could meaningfully extend this work. Beyond diagnostic classification, automated CA could be evaluated as a predictor of shunt responsiveness, building on recent randomized evidence of shunt efficacy in NPH. Longitudinal CA assessment on serial CT may help identify prodromal NPH at a stage where earlier intervention can improve long-term outcomes. While the callosal angle is a robust diagnostic marker, its univariate use has limitations. Combining automated callosal angle assessment with metrics like the Evans’ index, temporal horn width, and the ventricle-to-subarachnoid ratio may increase accuracy. Future clinical pathways are expected to integrate imaging with biochemical markers, such as amyloid-beta and tau levels in CSF, to stratify patients who may have mixed dementia. Finally, because head CT is more widely available and less costly than MRI, CT-based automated screening has the potential to expand access to NPH diagnosis in resource-limited settings.

### 4.2. Limitations

This study has several limitations. First, while the internal validation using 915 patients is one of the largest CT-based NPH cohorts to date, we acknowledge the lack of an independent, external test set. Performance metrics may therefore reflect optimization within our institution’s specific imaging protocols. Future studies utilizing multicenter data are required to validate the algorithm’s performance across the broader variability of clinical CT hardware and reconstruction kernels.

Second, while agreement between automated and manual CA measurements was high (ICC = 0.90), the 95% limits of agreement (±20.25°) were broader than those observed between manual raters (±10.40° to 12.10°). Although these wider limits may reflect greater differences at the extremes, because CA-based screening relies on thresholds such as 90–95°, this variance may still affect decisions for borderline cases. To mitigate this, automated CA values near diagnostic cutoffs could be automatically flagged for secondary radiologist review or evaluated in combination with additional imaging markers (e.g., ventriculomegaly ratios or NPH Radscale features) [2,14].

Third, this was a retrospective single-center study using CT data acquired on a limited set of scanners and reconstruction parameters. Automated CA performance may differ on scanners with distinct slice thicknesses, kernels, or noise characteristics. External multicenter validation will be necessary to confirm generalizability.

Fourth, no NPH reference standard is perfect; while we used multidisciplinary consensus rather than tap response alone, residual misclassification at the cohort level cannot be excluded.

Finally, although CA alone demonstrated strong diagnostic utility, it was not assessed for its prognostic value in predicting shunt responsiveness. Because the aim of this study was early screening rather than outcome prediction, this was beyond our scope, but correlating automated CAs with post-tap-test or post-shunt outcomes will be an important direction for future work.

### 4.3. Conclusions

Automated callosal angle measurement on routine head CT showed strong agreement with manual expert assessment and accurately distinguished patients with NPH from controls, with diagnostic performance comparable to MRI-based approaches. Because head CT is widely available and frequently obtained in the workup of older patients with falls, gait disturbance, or cognitive change, an automated tool of this kind could enable scalable opportunistic screening for a potentially reversible cause of dementia. External multicenter validation and integration with complementary imaging biomarkers represent the natural next steps toward clinical deployment.

## Figures and Tables

**Table 1:** Demographics.

Characteristic	NPH Patients	Control Patients
Number	198	198
Age (years)	74 ± 7	74 ± 7
Range (years)	54–94	54–94
25th/75th Quartiles (years)	70/79	70/79
Number of Men (frequency)	118 (60)	118 (60)
Number of Women (frequency)	80 (40)	80 (40)

**Table 2:** Diagnostic performance of manual and automated callosal angle measurements at different thresholds.

Method/Cutoff	Actual State	Predicted Control	Predicted NPH	Accuracy
Automated—Literature Cutoff 90°	Control	179	19	84.1%
	NPH	44	154	
Automated—Optimized Cutoff 95°	Control	170	28	85.9%
	NPH	28	170	

**Table 3:** NPH Radscale score counts for false negatives and positives observed for the automated measurement with an optimized cutoff of <95°.

	Radscale Score > 8	Radscale Score Between 4 and 8	Radscale Score < 4
False Negatives	5	20	3
False Positives	2	21	5

## Data Availability

The data that support the findings of this study are not publicly available due to privacy and ethical restrictions. The data consist of retrospective patient records from St. Joseph’s Hospital and Medical Center obtained with a waiver of informed consent for retrospective analysis and access is restricted to protect patient confidentiality.

## Abbreviations

The following abbreviations are used in this manuscript:

NPH	Idiopathic Normal Pressure Hydrocephalus
CA	Callosal Angle
MRI	Magnetic Resonance Imaging
CT	Computed Tomography
CSF	Cerebrospinal Fluid
ICC	Intraclass Correlation Coefficient
AUC	Area Under the Curve
ROC	Receiver Operating Characteristic
VP shunt	Ventriculoperitoneal Shunt
ICD	International Classification of Diseases
DESH	Disproportionately Enlarged Subarachnoid Space Hydrocephalus
MaxEccLV	Maximum Eccentricity of Lateral Ventricles (inferred from context, not explicitly defined)
ACR	American College of Radiology
AI	Artificial Intelligence
CI	Confidence Interval
